# Clearance of low levels of HCV viremia in the absence of a strong adaptive immune response

**DOI:** 10.1186/1743-422X-4-58

**Published:** 2007-06-11

**Authors:** Manuela F Meyer, Marc Lehmann, Markus Cornberg, Johannes Wiegand, Michael P Manns, Christoph Klade, Heiner Wedemeyer

**Affiliations:** 1 Department of Gastroenterology, Hepatology and Endocrinology, Hannover Medical School, D- 30623 Hannover, Germany; 2 Jugendanstalt Hameln, Tündernsche Straße 50, 31789, Hameln, Germany; 3 Clinical Immunology T-cell epitope Identifcation Program, Intercell AG, Campus Vienna Biocenter 6, 1030 Wien, Austria

## Abstract

Spontaneous clearance of hepatitis C virus (HCV) has frequently been associated with the presence of HCV-specific cellular immunity. However, there had been also reports in chimpanzees demonstrating clearance of HCV-viremia in the absence of significant levels of detectable HCV-specific cellular immune responses. We here report seven asymptomatic acute hepatitis C cases with peak HCV-RNA levels between 300 and 100.000 copies/ml who all cleared HCV-RNA spontaneously. Patients were identified by a systematic screening of 1176 consecutive new incoming offenders in a German young offender institution. Four of the seven patients never developed anti-HCV antibodies and had normal ALT levels throughout follow-up. Transient weak HCV-specific CD4+ T cell responses were detectable in five individuals which did not differ in strength and breadth from age- and sex-matched patients with chronic hepatitis C and long-term recovered patients. In contrast, HCV-specific MHC-class-I-tetramer-positive cells were found in 3 of 4 HLA-A2-positive patients. Thus, these cases highlight that clearance of low levels of HCV viremia is possible in the absence of a strong adaptive immune response which might explain the low seroconversion rate after occupational exposure to HCV.

## Background

Spontaneous clearance of acute infection with the hepatitis C virus (HCV) has been frequently associated with a strong HCV-specific cellular immune response [1,2]. Both HCV-specific CD4+ and HCV-specific CD8+ T cell responses were found to be stronger and longer lasting in chimpanzees and humans who cleared HCV as compared to individuals who developed a chronic course of the infection [3-13]. However, HCV-specific cellular immune responses were not always detectable in chimpanzees who cleared the virus spontaneously [14]. In addition, we and others have previously reported clearance of HCV in the absence of seroconversion to an anti-HCV antibody-positive status [15,16]. Subsequently, HCV-specific T cell responses in HCV-exposed anti-HCV-negative individuals have been demonstrated by several groups [17-20] although it was not known in the latter studies whether these subjects have transiently been viraemic for HCV or not. We had the chance to study prospectively individuals with acute hepatitis C in the setting of a German young offender institution. By systematic HCV-RNA screening of 1176 consecutive new incoming prisoners, we identified 7 HCV-RNA positive individuals who cleared HCV spontaneously during further follow-up. Four of those did never develop anti-HCV-antibodies. HCV-specific T cell responses could be monitored and were found to be rather weak and not significantly different from chronically infected individuals. Thus, these cases highlight that clearance of HCV-RNA may not necessarily correlate with the appearance of acquired immunity, a finding that challenges our current understanding of the immunopathogenesis of HCV-infection.

## Methods

### Patients

Details of the study cohort were presented previously [16,21]. In brief, all new incoming prisoners to the largest German young offender institution for young men (age 16–24) were screened for anti-HCV antibodies and HCV-RNA in the year 2002 (n = 1176). The study was approved by the Ethics Committee of the Hannover Medical School including additional blood sampling to study cellular immune responses.

Subjects tested positive for any of the HCV-markers (n = 97; 8.6% of individuals screened) were offered to participate in a follow-up study on the course of HCV-RNA, ALT levels and analysis of cellular immune responses. Follow-up data and PBMC of seven patients who had cleared HCV spontaneously were available for this study. In four of them, intravenous drug abuse was the most likely source of infection. Three patients reported unsafe sex before imprisonment as the only risk factor possibly being associated with HCV infection. Characteristics of the seven subjects with cleared HCV-infection are shown in table 1. Thirteen patients with chronic HCV infection (HCV-RNA-positive at baseline and after 3 and 6 months of follow-up) and 9 individuals with resolved HCV-infection (anti-HCV-positive/HCV-RNA-negative at baseline and after 3 and 6 months of follow-up) identified in the same screening served as controls for the T cell assays. Characteristics of control patients are shown in tables 2 and 3. Control subjects did not differ in age (20.0 ± 1.4 years and 20.7 ± 1.4 years for patients with persistent infection and individuals being HCV-RNA-negative already at baseline) to the seven study subjects (20.1 ± 0.8 years). All individuals studied were male. None of the subjects had received antiviral therapy for HCV-infection prior to incarceration or after imprisonment.

**Table 1:** Characteristics of Cases with spontaneous HCV clearance

**Subject #**	**Age (Years)**	**Risk factor**	**Duration of IVDU (months)**	**HLA class I**	**Peak ALT (U/L)**	**HCV Geno-type**	**Peak HCV viremia (IU/ml)**	**Anti-HCV-antibodies ELISA**	**Immuno- Blot (Inno-Lia)**
1	19	Unsafe sex	n.a.	A03,11;B07,50; Cw06,07	17	1	1 × 10^3^	Negative	Negative
5	20	Unsafe sex	n.a.	A01; B08; Cw07	21	1a	1 × 10^4^	Negative	Negative
10	19	IVDU	18	A02;B15,39; Cw03,07	8	1b	3 × 10^2^	Positive	Positive (6 proteins)
13	21	Unsafe sex	n.a.	A02,24;B07,44; Cw04,07	22	1	7 × 10^3^	Negative	Negative faint band*
27	21	IVDU	72	A01,03;B35,57; Cw04,06	12	1a	1 × 10^4^	Positive	Positive (4 proteins)
36	20	IVDU	10	A02,24;B42,35; Cw04,17	25	1	5 × 10^4^	Negative	Negative
80	21	IVDU	24	A02,68;B14,44; Cw07,08	250	1**	Pos.	Positive	Positive (4 proteins)

(*) Patient 13 had a faint band against the C2 protein at the second time point investigated (after HCV clearance).

(**) Patient 80 had HCV serotype 1. Genotyping was not performed in the initial viremic sample and thus the HCV serotype was determined from HCV-RNA-negative follow-up samples.

n.a.: not applicable

**Table 2:** Characteristics of control patients with chronic hepatitis C

**Subject #**	**Age (Years)**	**Risk factor**	**Duration of IVDU (months)**	**Peak ALT (U/L)**	**Peak HCV viremia (IU/ml)**	**Anti-HCV-antibodies ELISA**	**Genotype**
4	18	IVDU	6	168	1 × 10^6^	Positive	3
12	18	IVDU	27	34	2 × 10^5^	Positive	2
14	19	IVDU	18	44	2 × 10^6^	Positive	1
29	20	IVDU	36	21	2 × 10^6^	Positive	1
31	18	IVDU	72	715	9 × 10^6^	Positive	1
32	20	IVDU	24	57	3 × 10^6^	Positive	3
44	21	IVDU	84	49	4 × 10^5^	Positive	2
48	22	Unsafe Sex	n.a.	13	4 × 10^5^	Positive	1
56	22	IVDU	60	193	1 × 10^6^	Positive	1
57	22	IVDU	36	34	2 × 10^6^	Positive	1
69	21	IVDU	36	94	1 × 10^4^	Positive	3

**Table 3:** Characteristics of control patients who were anti-HCV+/HCV-RNA negative at incarceration and during follow-up of 6 months.

**Subject #**	**Age (Years)**	**Risk factor**	**Duration of IVDU (months)**	**Peak ALT (U/L)**	**Anti-HCV-antibodies ELISA**	**HCV-RNA**
15	22	Unsafe sex	n.a.	19	Positive	Negative
30	22	n.a.	n.a.	11	Positive	Negative
35	20	IVDU	24	29	Positive	Negative
40	20	n.a.	n.a.	11	Positive	Negative
50	21	IVDU	24	10	Positive	Negative
59	20	n.a.	n.a.-	6	Positive	Negative
60	19	IVDU	10	55	Positive	Negative
71	21	IVDU	2	16	Positive	Negative
94	21	IVDU	24	321	Positive	Negative

### Serologic and virologic testing

Serum samples were tested for anti-HCV-antibodies using a third generation HCV-ELISA (AxSYM-HCV Version 3.0, Abbott Diagnostics, Wiesbaden, Germany). Antibody reactivity against single HCV antigens was evaluated using the immunoblot assay INNO-LIA HCV III Update (Innogenetics, Ghent, Belgium). HCV-RNA testing was performed by Real-Time PCR (sensitivity 10^2 ^copies/ml) as previously described [16,21]. For low viremic samples, we always performed a second RNA extraction and applied a second PCR with an alternative primer set and thus positive results in this study were based on at least two independent extractions and two independent PCRs. The HCV-RNA PCR has been validated and is used as a routine diagnostic method in laboratory of the NLGA (Niedersächsisches Landesgesundheitsamt) which is the central laboraty of public health department of the state of Lower Saxony. The frequency of HCV-RNA positive results in anti-HCV-negative samples was always far below 1%.

### T cell assays (Hannover Lab)

HCV-specific CD4+ T cell responses were investigated by interferon-gamma ELISPOT-assays and proliferation assays as described [22,23]. Antigens (HCV-core, HCV-NS3, HCV-helicase, HCV-NS4, HCV-NS5a, tetanus toxoid) were purchased from Mikrogen (Munich, Germany). Cyropreserved PBMC were used in all experiments. To avoid inter-assay variability, all serial samples follow-up of one individual were tested in the same assay. A stimulation index (SI) of >2.5 in the proliferation assay was considered positive (the mean SI in healthy controls + 3 standard deviations was <2.5 for all proteins tested). In the ELISPOT-assay, a positive result was considered if spot-forming units in the presence of antigen were at least double of spot forming units in the medium control and if at least five spots were detected per well.

Since patients recruited at the young offender institution in Hameln had a high risk of potentially being exposed to HCV due to previous drug consumption and HCV-specific T cell responses have frequently been shown in seronegative HCV-exposed individuals (including our own experience showing induction of HCV-specific T cell responses after needle stick injury), we did not choose individuals from the young offender institution as a "negative" control group but applied identical methods as for the other projects run in the laboratory. T cell responses were investigated in the laboratory in several different cohorts of persistently apparently unexposed HCV-RNA-negative individuals showing frequencies of responses for the different assays of <5% for CD4+ T cell responses [23-25].

Positive controls were run in each assay. We had control samples from patients with symptomatic acute hepatitis C recruited in the Hep-Net acute HCV-II study [26] with a robust HCV-specific CD4+ and CD8+ T cell response. Our data on T cell responses of patients treated in the Hep-Net acute HCV-II study have been described elsewhere [25].

HCV-specific CD8+ T cell responses were tested only in HLA-A2-positive subjects since MHC-class I-HCV-tetramers were available only for HLA-A2-restricted epitopes (Table 4). HCV-tetramers were purchased from ProImmune (Oxford, UK). PBMC (4 × 10^6 ^cells/ml) were washed once with 0,1% BSA 0,1% sodium acide in PBS and incubated seperately with four HLA-A2 restricted, HCV-specifiv tetramers (all HLA*0201; Core-132 DLMGYIPAV; NS3-1073 CINGVCWTV; NS3-1406 KLVALGINAV; NS5 ALYDVVTKL) for 20 min at 37°C. 1 μl of antibodies (anti-CD8 APC, anti-CD27 FITC, anti-CD28-FITC, anti-CD69 Cy, anti-CD45 RO FITC/PE (Beckdon Dickinson, Heidelberg, Germany) were added for 10 min at RT. After two washing steps, cells were analysed by flow cytometry (FACS Calibur, Beckdon Dickinson, Heidelbeg, Germany). 1 × 10^5 ^cells in the lymphogate were collected for each analysis. Data were aquired with CELLQUEST program (Beckdon Dickinson, Heidelbeg, Germany). In addition, interferon-gamma ELISPOT-responses to a panel of 8 selected peptides were tested as described [23].

**Table 4:** Sequences of HLA-A2-restricted peptides used in ELISPOT assays

Region	Sequence	Tetramer?
Core-35	YLLPRRGPRL	No
Core-132	DLMGYIPLV	Yes
Core-178	LLALLSCLTV	No
NS3-1169	LLCPAGHAV	No
NS3-1073	CVNGVCWTV	Yes
NS3-1406	KLVALGINAV	Yes
NS5-2594	ALYDVVTKL	Yes

### ELISPOT assays (Vienna Lab)

Cryopreserved PBMC were also shipped to a second lab in Vienna headed by CK. GCP-validated ELISPOT assays are performed in this laboratory on a routine bases for measuring T cell responses in clinical vaccine trials [27]. Assays were performed exactly as previously described [28]. Since not enough PBMC were available to screen for T cell responses with overlapping peptides, a panel of synthetic peptides representing ~30 MHC class I and class II T cell epitopes derived from conserved regions of HCV was used (Table 5). These peptides were identified in a systematic screening with >400 overlapping peptides in PBMC from 65 HCV-recovered individuals (Klade et al., manuscript in preparation). The 30 selected peptides have a cumulative HLA coverage > 85%.

**Table 5:** Sequences of peptides used for the ELISPOT-screen in the Vienna laboratory at Intercell AG.

	**HCV**		**HLA coverage**	
**ID**	**antigen**	**amino acid sequence**	**class I**	**class II**
1798	NS4	IGLGKVLVDILAGYGAGVAGALVAFK	A2, 3, 11	DR1, 4, 7
1799	NS3	AAWYELTPAETTVRLR	B7/B35	DR1, 4
1624	E2	LEDRDRSELSPLLLSTTEW	B*4001	DR7
1547	NS3	YLVAYQATVCARAQAPPPSWD	A2	DR1, 4, 7, 11
1827	NS3	TAYSQQTRGLLG	A24, B8?	DR1, 7, 11
1829	NS5	SMSYTWTGALITP	A2, B7, A24?	DR1, 7, 11?
1846	E2	DYPYRLWHYPCTVNFTIFKV	Cw7, A2, A11?	DR1, 4, 7, 11
1754	E2	DYPYRLWHY	Cw7	
1835	Core	KFPGGGQIVGGVYLLPRRGPRLGVRATRK	A2, A3, B7	DR11
1855	NS5	SAKSKFGYG	B8?	
1843	Core	LPRRGPRL	B7	
1844	Core	GPRLGVRAT	B7	
1818	NS3	TPAETTVRL	B7/B35	
1838	NS4	SPGALVVGVI	B7	
1557	NS5	SSMPPLEGEPGDPDL	B*4402?	

## Results

### Cases

**Figure 1:**
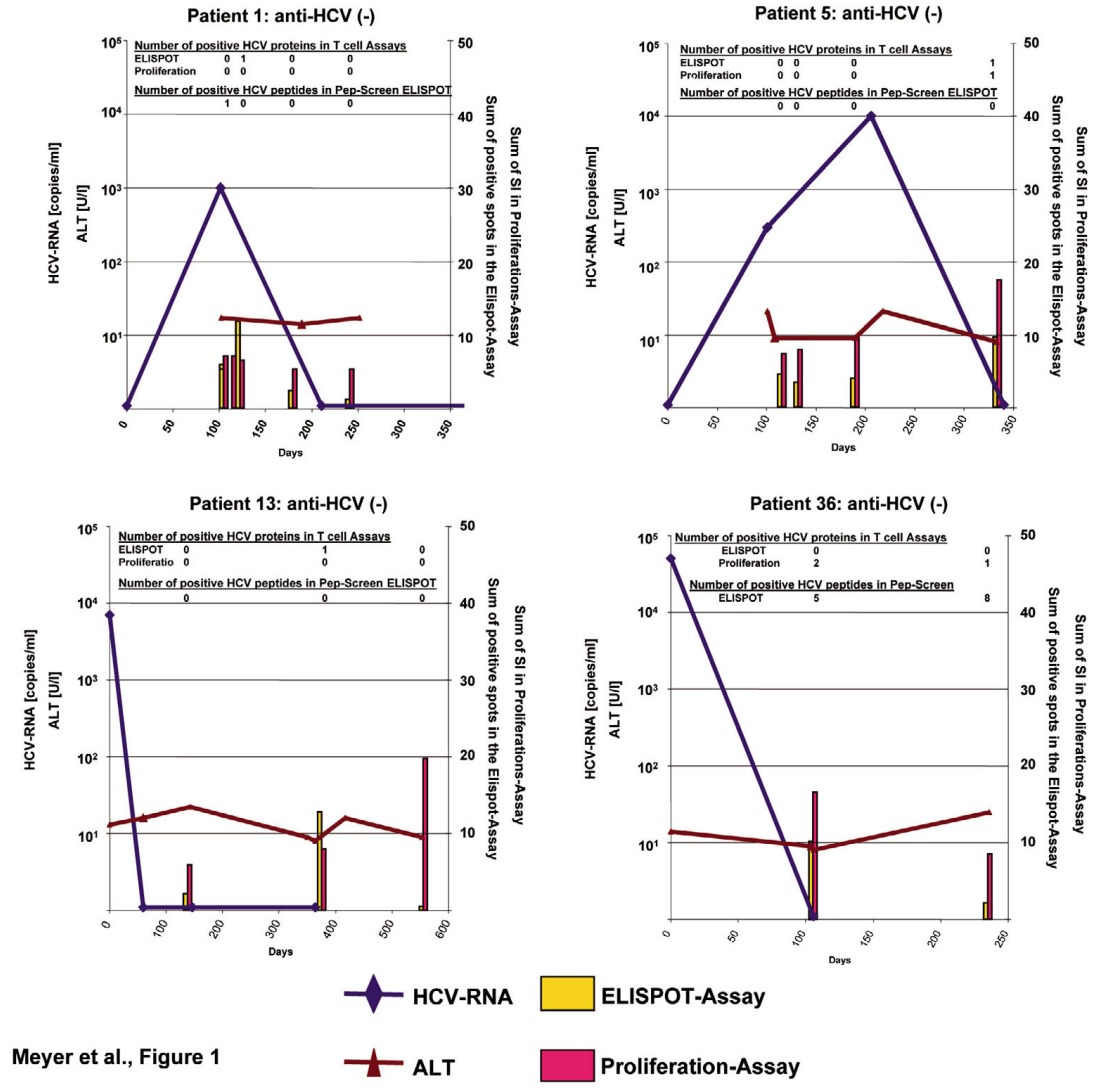
Biochemical, virological and immunological course of acute hepatitis C in asymptomatic patients with spontaneous clearance: patients with spontaneous clearance in the absence of anti-HCV. HCV-RNA levels, ALT levels and T cell responses are shown. Coloured bars show the sum of SI values of the proliferation assay and the sum of specific interferon-gamma spots (spots in the presence of antigen – spots in the medium control) against 5 individual HCV proteins (HCV-core, HCV-NS3, HCV-helicase, HCV-NS4, HCV-NS5a). In addition, the number of individual proteins tested positive at the respective time point is shown for each assay. Interferon gamma ELISPOT responses were also tested against a panel of synthetic peptides representing class I and class II T cell epitopes derived from conserved regions of HCV with a cumulative HLA coverage > 85%. These assays were performed in an independent second laboratory ("Pep-Screen ELISPOT"; Vienna lab). The number of peptides tested positive in this assay is also given.

Out of 97 subjects identified to be anti-HCV-positive and/or HCV-RNA-positive during the initial screening after incarceration, 90 individuals agreed to participate in the follow-up study and were studied after 3 and 6 months for HCV-RNA and ALT levels. At baseline 71 were HCV-RNA-positive and 19 HCV-RNA-negative [16]. Seven of the HCV-RNA-positive patients cleared HCV-RNA spontaneously after 8–31 weeks of follow-up. Characteristics of these seven patients are shown in table 1 and the time course of HCV viremia, ALT levels and T cell responses is shown in figure 1. Only one subject (#80) had biochemical evidence of hepatitis with ALT levels higher than two times upper the limit of normal. None of the individuals reported significant symptoms associated with hepatitis or developed jaundice. Liver function as determined by albumin levels and prothrombine time remained normal in all subjects at any time. Bilirubin levels were minimally elevated once in patient #1 (1.23 mg/dl; normal value 1.1 mg/dl), and at three time points in patient #5 with levels between 1.5 and 2.3 mg/dl. Mean peak HCV-RNA levels were lower in the seven cases with spontaneous HCV clearance than in 62 HCV viraemic patients who did not recover from HCV (1.1 × 10^4 ^± 1.6 × 10^4 ^copies/ml vs. 2.0 × 10^6 ^± 3.8 × 10^6 ^copies/ml for patients with HCV clearance and patients with persistent infection, respectively). None of the seven patients was co-infected with HIV or HBV.

### Humoral Immunity

Anti-HCV antibodies as determined by a standard 3^rd ^generation ELISA-assay remained negative throughout follow-up in 4 of the 7 seven cases (Table 1). We also investigated reactivity against single HCV proteins by the INNO-LIA HCV III assay. A faint band against the C2 protein was found once in one of the four anti-HCV negative patients while all anti-HCV-positive patients tested positive for at least four HCV-antigens at all time points (Table 1).

### HCV-specific CD4+ T cell responses

**Figure 2:**
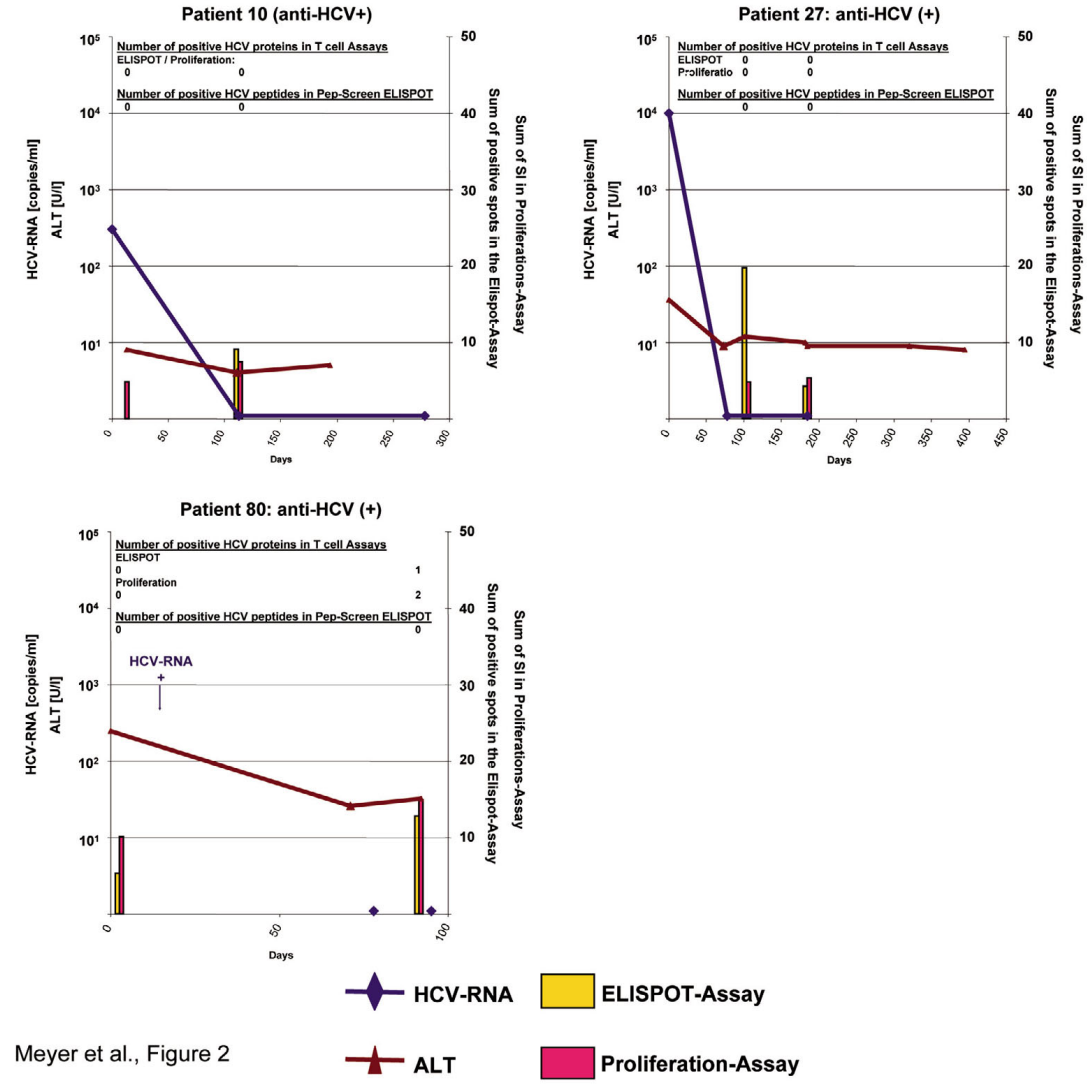
Biochemical, virological and immunological course of acute hepatitis C in asymptomatic patients with spontaneous clearance: patients who developed anti-HCV antibodies.

Weak transient HCV-specific CD4+ T cell responses were detected against at least one HCV protein either in the interferon-gamma ELISPOT assay or in the proliferation assay in five of the seven patients (figures 1 and 2). None of the 4 patients who remained negative in the anti-HCV ELISA (#1, #5, #13, and #36) had a CD4+ response that was detectable at more than one time-point (figure 1). Moreover, in all but one case (patient #36, first timepoint investigated) only one protein tested positive in the anti-HCV-negative patients.

Anti-HCV antibodies were already present at baseline in patients #10 and #27 (figure 2). No HCV-specific CD4+ T cell responses were found before or after HCV clearance in these two patients. Subject #80 was the only individual with biochemical evidence of hepatitis with about ten times elevated ALT levels. A robust response against HCV-helicase was found in the proliferation assay at baseline and after clearance of HCV-RNA (SI values of 4.1 and 3.6, respectively). At the second time point, we found also a significant response against NS4 in the proliferation assay as well as a helicase-specific interferon gamma response in the ELISPOT assay detectable (figure 2).

Overall samples from 19 time points were investigated in the 7 patients with spontaneous HCV clearance (2–4 time points per patient as shown in figure 1). At least one HCV protein was reactive in 4 (22%) of the ELISPOT-assays and 3 (16%) of the proliferation assays (table 6). In 7 cases (37%) any of the two CD4 assays yielded at least one positive result. Comparing these results with ELISPOT assays from control subjects from the same cohort with chronic hepatitis C did not show any significant differences in frequency, breadth and strength of CD4+ T cell responses. Interferon gamma-ELISPOT responses were found in 6/24 assays (25%) performed in 13 subjects with chronic HCV viremia. The mean number proteins recognized per assay did also not differ between the two groups (table 6). Five of the 13 chronically infected patients (38%) mounted at least one positive interferon gamma response. The absolute number of spot forming units in the ELISPOT-assay in positive cases was not different in patients who had cleared HCV and in chronic hepatitis C patients (mean SFU 16.8 ± 1.1/10^6 ^PBMC for patients with clearance and 19.4 ± 4.8/10^6 ^PBMC for chronic HCV patients). HCV long-term-recovered control patients (anti-HCV positive and HCV-RNA-negative already at baseline) showed ELISPOT responses in 5/12 assays and proliferative responses in 3/9 assays performed with samples from 9 individuals also with a similar strength in responses (table 6).

**Table 6:** Summary of HCV-specific T cell responses obtained with different assays in the three different groups of patients studied

		Proliferation Assay		Class II ELISPOT assay		Class I Tetramer Assay	
	Number of patients	Time points with at least 1 positive protein	proteins tested positive/time point (mean number)	Time points with at least 1 positive protein	proteins tested positive/time point (mean number)	Time points with at least 1 positive tetramer	tetramers tested positive/time point (n)
Index patients (Acute resolving hepatitis C)	7	3/19 (16%)	0.26	4/19 (22%)	0.42	5/9 (56%)*	1.8
Chronic hepatitis C	13	2/10 (20%)	0.20	5/20 (25)	0.30	-	-
Anti-HCV+/HCV-RNA-	9	3/9 (33%)	0.44	5/12 (42%)	0.42	-	-

* Only 4 pts were HLA-A2 pos

### HCV-specific CD8+ T cell responses

HCV-specific CD8+ T cell responses were investigated by MHC-class I tetramer stainings in the HLA-A2-positive patients. Patient #13 (anti-HCV-negative) displayed significant responses for three HCV tetramers with frequencies between 0.08% and 0.13% of CD8+ T cells at the second time point (more than 6 months after HCV clearance). The patient tested negative with the tetramer assay at the two other time points (both after HCV clearance). He also showed interferon gamma responses in the ELISPOT assay against five individual HCV peptides at the first time point but not thereafter during follow-up. Patient # 36 (anti-HCV-negative) was negative in both assays applied to investigate CD8+ T cell responses at both time points when PBMC were available.

Patient # 10 (anti-HCV-positive) had the highest frequency of HCV-tetramer-positive cells at baseline with 0.31% Core-132-specific, 0.99% NS3-1073-specific, 0.40% NS3-1406-specific and 0.4% NS5-specific tetramer-positive CD8+ T cells. The frequencies of peripheral HCV-specific CD8+ T cells significantly declined as compared with the second time point when PBMC were available 3 months later. At this time, only HCV-NS3-1073-specific T cells remained detectable with 0.11% of CD8+ T cells. Patient #80 (the only patient with biochemical evidence of hepatitis) had robust frequencies of NS3-1073-specific T cells with 0.22% of CD8+ T cells at both time points investigated. The other tetramers gave only borderline responses with 0.05%-0.10% of CD8+ cells. After clearance of HCV, interferon-gamma ELISPOT responses were found against 4 peptides including the NS3-1073 and core-132 in this subject.

### IFN-gamma responses against 30 class I and II epitopes (Vienna lab)

**Figure 3:**
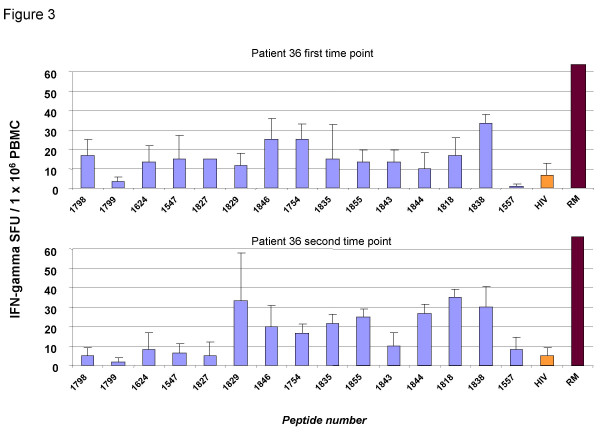
Peptide screen in patient 36. Only subject #36 mounted a robust multispecific response against at least 5 peptides after clearance of HCV at two independent time points (figure 2). Patient #1 tested positive for one HCV-class II peptide at the first time-point investigated and was negative for all peptides on subsequent time points. Patients #5, #10, #13, #27, and #80 tested negative for all peptides at all time-points investigated. All other patients tested negative in this assay.

Since only a limited number of PBMC was available according to the study protocol approved the institutional ethics committee, it was not possible to apply a full breath screening for T cell responses with overlapping peptides. Thus, we used a panel of 30 synthetic peptides representing ~30 class I and class II T cell epitopes derived from conserved regions of HCV with a cumulative HLA coverage > 85%. These peptides were tested in an independent second laboratory. Also in this T cell readout most patients tested negative at all time points investigated. Patient #1 tested positive for one HCV-class II peptide at the first time-point investigated and was negative for all peptides on subsequent time points. Patients #5, #10, #13, #27, and #80 tested negative for all peptides at all time-points investigated. Only subject #36 mounted a robust multispecific response against at least 5 peptides after clearance of HCV (figure 3) which were not HLA-A2-restricted and thus no tetramer assays could be performed in this study for this individual.

## Discussion

We here report seven cases of spontaneous clearance of hepatitis C virus identified through a systematic screening of young male subjects with a high proportion of iv-drug addicts. We believe that the cases are of special interest for several reasons:

(i) Four individuals never developed anti-HCV antibodies in a standard 3^rd ^generation ELISA-assay and also tested negative in an anti-HCV immunoblot assay. Thus, without screening for HCV-RNA these subjects would never have been recognized as being infected with HCV. Similar cases of HCV clearance in the absence of anti-HCV seronconversion have recently been described in four Australian IV-drug addicts [15]. In line with reports on HCV-specific T cell responses in seronegative HCV-exposed persons [17-20], it is very likely that the true rate of individuals in the general population who had contact with HCV is underestimated, in particular in high risk persons such as iv-drug addicts and maybe also in medical health professionals. Our systematic unbiased screening approach of all new incoming prisoners in a young offender institution [16] supports the conclusion that the percentage of patients who are able to clear HCV viremia without developing anti-HCV antibodies might be much higher than previously thought since the anti-HCV ELISA became positive in only three out seven patients.

(ii) HCV was cleared spontaneously even though the subjects were asymptomatic and six out of seven had no biochemical evidence of hepatitis. On the first view, these findings somewhat contradict with several previous reports suggesting that the likelihood to clear HCV spontaneously is the higher the more symptomatic a patient is and the higher liver transaminases are [29,30]. We have no information how many patients with similar characteristics of exposure have developed chronic infection in our setting since the subjects were identified in a cross-sectional screening approach. At least 65 initially HCV-RNA positive patients had persistent infection although we do not know when these subjects had been infected before incarceration [16]. Moreover, characteristics of our patients differ from most other studies on acute hepatitis C. Our patients were rather young with an age between 16 and 24 and the mode of infection was iv drug abuse in almost all cases. Peak HCV-viral load with levels between 3 × 10^2 ^and 5 × 10^4 ^copies/ml was 2–3 logs lower than in patient cohorts on acute hepatitis C that were enrolled in treatment trials [26,31,32]. Nevertheless, for the management of acute hepatitis C patients our study supports the concept to investigate the kinetic of HCV-viremia for 2–3 weeks before an antivirial therapy is considered [33] – at least for individuals with a viremia of < 5 × 10^5 ^IU/ml. If HCV-RNA declines, there might be a high chance for spontaneous clearance even in asymptomatic patients.

(iii) HCV-specific CD4+ T cell responses were rather weak or even undetectable and all subjects cleared HCV anyway. Clearance of HCV has been associated with strong and multispecific cellular immune responses in chimpanzees and humans [2]. Our findings are not completely in line with this dogma but are supported by at least one report on chimpanzees where also no correlation of HCV-specific adaptive immunity with HCV clearance was evident [14]. In our study, HCV-specific CD4+ responses were completely undetectable in 2 subjects and rather weak in the remaining 5 individuals despite clearance of HCV. These responses did not differ in strength and specificity from chronically infected control subjects. Of note, identical methods were applied and even the same proteins were used for the in vitro stimulation of T cells in the different assays as in our own previous studies [23-25,34,35] and also as in studies from other laboraties. Moreover, the low frequency and strength of T cells in our assays could not be explained by a mismatch of HCV proteins used for stimulation in the in vitro assays with the HCV genotype infecting the patients since all subjects had been infected with HCV genotype 1. In contrast, HCV-specific CD8+ T cell cells were found by tetramer staining in three of the four patients, although the frequencies of tetramer-positive cells were rather low in 2 of them as they did not exceed 0.2% of CD8+ T cells which was much lower as compared to our own experience on symptomatic acute hepatitis C patients [25] and also as compared to other reports on human subjects who cleared HCV spontaneously [8-10,12]. Nevertheless, the finding that three out of four HLA-A2 positive patients displayed HCV-specific CD8+ suggests that indeed T cell responses have been primed and that CD8+ T cells may have contributed to clearance of HCV. Importantly, the overall low frequency of HCV-specific T cells in the subjects investigated here was confirmed by T cell testing in a second, independent laboratory using a GCP-validated ELISPOT assay used for investigation of PBMC in clinical trials [28].

What could be the reasons for the lack of an apparent association between HCV-specific CD4+ T cell responses and recovery from HCV? First, our data suggest that low levels of HCV viremia as found in our seven cases may be controlled by innate immune responses without a strong adaptive immunity. Data derived from chimpanzees indeed have demonstrated that early intrahepatic induction of interferon-response genes without significant recruitment of HCV-specific T cells leads to significant reduction of HCV viral load [5]. Moreover, also Post et al. described a rather low strength of T cell responses in their two subjects with HCV clearance in the absence of seroconversion [15]. However, since we had not enough PBMC available to apply an overlapping peptide approach in our patients we might have missed some CD4+ and very likely some CD8+ T cell responses although the second screening with a panel of 30 peptides covering >85% of HLA types should have picked most responses. Nevertheless, the overall strength of responses might have been underestimated in our study. However, the important message is that the strength and frequency of T cell responses in patients with spontaneous clearance was not different from chronically infected or long-term recovered patients.

Also for HIV infection it has been shown that an antigenic threshold for maintaining cytotoxic T cell responses may be required [36]. The required levels of antigenic stimulation may not have been reached in our patients but innate immune responses may have led suppression of HCV replication. We also do not know, if our patients had been exposed to HCV before. Four of the seven patients were IV drug addicts with a duration of abuse of 18–72 months before incarceration. Higher rates of HCV clearance in iv-drug addicts have been reported. However, previous contact to HCV and re-exposure should have led to an expansion of adaptive immune responses which were not detectable in our patients. The lower HCV-RNA levels in our patients may be also explained by the route of exposure since three out of four subjects who did not develop anti-HCV antibodies had reported sexual exposure while almost all chronically infected patients were IV drug addicts.

For five of the seven patients we do not know how long these individuals had been viremic prior to imprisonment and thus we may have missed peak HCV-RNA levels and also some adaptive immune responses occurring prior to imprisonment. However, the main message of this manuscript is that HCV was cleared in patients without the development of anti-HCV antibodies in 4 out of 7 individuals. Moreover, CD4+ T cell responses are usually maintained for some time after clearance if no spontaneous relapse occurs as shown already in 1999 by Gerlach et al. [37].

Homing of T cells to the liver has been described in chimpanzees during acute HCV infection [5]. It is not possible to perform liver biopsies in patients with acute hepatitis C since there is no clinical indication for this invasive procedure. Thus, we can not exclude the presence of stronger intrahepatic HCV-specific T cells in our patients.

One could question if subjects #1, #13 and #36 indeed had acute hepatitis C since HCV-RNA tested positive only once in the absence of anti-HCV and thus the HCV-PCR could have been false-positive. However, a positive HCV-PCR result was always double checked and investigated by both qualitative and quantitative PCR. Moreover, Patient #36 mounted a significant and broad HCV-specific cellular immune response (figure 1A and 2) supporting contact to the hepatitis C virus. Patient #13 had a significant peak viremia of 7.000 copies/ml making a false positive PCR result due to contamination rather unlikely and this patient also showed HCV-specific CD8+ T cells by tetramer staining.

In conclusion, clearance of HCV-RNA may not necessarily correlate with the appearance of acquired immunity. This finding challenges our current understanding of the immunopathogenesis of HCV-infection and maybe an explanation for the rather low seroconversion rate after occupational exposure to HCV. The data should also be considered in the management of accidental findings of low levels HCV viremia in asymptomatic individuals which may have a higher chance of spontaneous clearance than previously thought.
